# Children and adolescents with DiGeorge syndrome are short in early infancy but develop excess weight in adolescence: a retrospective study

**DOI:** 10.1590/1984-0462/2025/43/2024311

**Published:** 2025-12-01

**Authors:** Renata Gomes de Oliveira, Marina Benevides Pinheiro Cavalcante, Laura Dresch Neumann, Davi Casale Aragon, Pérsio Roxo, Fabio Carmona

**Affiliations:** a Universidade de São Paulo, Faculdade de Medicina de Ribeirão Preto, Ribeirão Preto, SP, Brasil.

**Keywords:** 22q11 deletion syndrome, Primary immunodeficiency diseases, Body weight, Height, Body mass index, Growth, Síndrome da deleção 22q11, Doenças da imunodeficiência primária, Peso corporal, Estatura, Índice de massa corporal, Crescimento

## Abstract

**Objective:**

DiGeorge syndrome (DGS) is characterized by facial dysmorphisms, congenital heart defects, palatal abnormalities, hypocalcemia, immunodeficiency, and neurodevelopmental deficits. Growth hormone deficiency may be present. Among these, recurrent infections, congenital heart defects, hypocalcemia, and feeding difficulties are the most common causes of impaired growth. The aim of this study was to describe and compare over time the growth trajectory of children and adolescents with DGS followed up in an outpatient clinic at a Brazilian tertiary-care referral hospital.

**Methods:**

In this historical cohort, we analyzed electronic medical records of 29 patients collected from 2009 to 2024.

**Results:**

In the first 2 years of life, 50% of weight measurements were below a z-score of -2 (indicating low or very low weight for age). Between 2 and 10 years of age, the median weight was between z-scores -2 and 0 (appropriate weight for age), and there was a significant increase in weight/age over time (p=0.019). Regarding length, in the first 2 years of age, 75% of the measurements were below a z-score of -2. From 2 to 19 years of age, the median length was between z-scores -2 and 0, suggesting recovery over time (p=0.004). Between 2 and 10 years of age, the median body mass index (BMI) was between z-scores -2 and 0 (normal weight). After 11 years of age, the median BMI-for-age z-score was above +1 (excess weight). Over time, there was a significant increase in BMI-for-age z-scores (p=0.011).

**Conclusions:**

Weight and height deficits were prevalent in the early years, likely associated with feeding difficulties and congenital defects, but showed recovery around 2 years of age. Recognizing anthropometric variations in patients with DGS helps prioritize interventions such as nutritional support, infection control, and surgical correction.

## RESUMO

**Objetivo:**

A síndrome de DiGeorge inclui dismorfismos faciais, defeitos cardíacos congênitos, anomalias palatais, hipocalcemia, imunodeficiência e déficits do neurodesenvolvimento. Pode haver, também, deficiência do hormônio do crescimento. Infecções recorrentes, cardiopatias, hipocalcemia e dificuldades alimentares são as causas mais comuns do comprometimento do crescimento. Este estudo visa a descrever o crescimento de crianças e adolescentes com síndrome de DiGeorge acompanhados em hospital terciário de referência brasileiro.

**Métodos:**

Estudo de coorte histórico, analisando 29 pacientes atendidos entre 2009 e 2024.

**Resultados:**

Nos 2 primeiros anos de vida 50% das medidas de peso estavam abaixo do escore z -2 (baixo e muito baixo peso para a idade). Entre 2 e 10 anos a mediana do peso ficou entre z -2 e 0 (adequado para a idade), e houve aumento significativo do peso/idade ao longo do tempo (p=0,019). Em relação ao comprimento, nos 2 primeiros anos, 75% estavam abaixo do escore z -2. De 2-19 anos, a mediana ficou entre escore z -2 e 0, sugerindo recuperação (p=0,004). A mediana do Índice de Massa Corporal (IMC) ficou entre os escores z -2 e 0 (peso normal) até os 10 anos. Após 11 anos, a mediana do escore z ficou acima de +1 (excesso de peso). Ao longo do tempo houve aumento significativo do IMC (p=0,011).

**Conclusões:**

Déficits de peso e altura foram prevalentes nos primeiros anos, provavelmente associados a dificuldades de alimentação e defeitos congênitos, recuperando-se em torno dos dois anos. Reconhecer variações antropométricas nesses pacientes ajuda a priorizar intervenções como suporte nutricional, controle de infecção e correção cirúrgica.

## INTRODUCTION

DiGeorge syndrome (DGS) was first described by Kirkpatrick and DiGeorge in 1968.^1^ It is characterized by a triad of cardiac anomalies, thymic hypoplasia, and hypocalcemia,^2^ with an estimated prevalence of 1 in 3000–6000 live births, occurring equally in both sexes.^3^ Its variable phenotype includes facial dysmorphisms, airway malformations, dental and ophthalmological abnormalities, neurodevelopmental disorders, immune system defects, and a predisposition to neoplasms. Immunological changes are present in a variable spectrum, including T lymphopenia, deficient cytokine production, reduced T lymphocyte proliferation, and hypogammaglobulinemia.^4^ 22q11.2 deletion syndrome (22q11.2DS) is a genetic condition caused by the loss of a small portion of chromosome 22, specifically in the q11.2 region. This deletion can lead to a wide range of clinical manifestations, which vary significantly among affected individuals. The main features include congenital heart defects, palatal anomalies, immune deficiencies, distinctive facial features, and learning difficulties. Additionally, hearing, ocular, skeletal, and genitourinary abnormalities may occur, along with psychiatric and autoimmune disorders. The syndrome follows an autosomal dominant inheritance pattern, although most cases result from *de novo* deletions.^5^

Patients with inherited immune disorders may fail to thrive, and the potential long-term consequences of this failure are not fully understood. In Chile and Italy, patients with SDG gain insufficient weight in early childhood, experience catch-up in the following years, and become overweight or obese in adolescence and adulthood,^6,7^ particularly among women.^6,7^ However, there are no studies on the prevalence or the growth of patients with SDG in Brazil, a culturally and economically diverse country.

Thus, the primary objective of this study was to describe and compare over time the growth trajectory (weight, length/height, and body mass index [BMI]) of patients with DGS followed up in an outpatient clinic at a Brazilian tertiary-care referral hospital.

## METHOD

This was an electronic medical records-based retrospective study (historical cohort) that adheres to the REporting of studies Conducted using Observational Routinely-collected health Data (RECORD) Statement.^8^ This study is a secondary analysis of data from another study, previously approved by the Research Ethics Committee of the Ribeirao Preto Medical School, University of Sao Paulo (CAAE 76871823.8.0000.5440), in which some participants signed an informed consent form (those requiring a blood draw) and some did not (waived by the institutional review board [IRB]).

Patients of both sexes, aged 1–19 years, seen at the Immunodeficiencies Outpatient Clinics of Hospital das Clínicas de Ribeirão Preto (HCRP) between 2009 and 2024, and with a confirmed genetic diagnosis of DGS, were eligible. Patients with other innate immune errors or associated genetic syndromes were excluded. The medical records of all eligible patients were reviewed, and the following data were collected: demographics (age and sex); presence of heart disease, hypocalcemia, and thymus; genetic testing results (multiplex ligation-dependent probe amplification [MLPA], fluorescence in situ hybridization [FISH], others); chronic oral corticosteroid use; immunoglobulin replacement therapy; feeding route (oral or enteral); birth weight; and anthropometric measurements (weight and length/height) recorded by nursing staff according to standard procedures.

Weight and length/height values were initially reviewed for implausible values and unit corrections. Subsequently, BMI was calculated whenever weight and length/height were available. Z-scores for weight-for-age (W/A), weight-for-length (W/L), length/height-for-age (LH/A), and BMI-for-age (BMI/A) were calculated according to the standard^8^ or the reference growth curves^9^ using the WHO Anthro software (WHO, Geneva, Switzerland) or the *zanthro* package for StataSE 14.0 software (StataCorp, College Station, USA). The z-scores were categorized by age groups according to the Weffort and Silva:^10^ infants (0–2 years), preschoolers (2–6 years), schoolers (7–10 years), and adolescents (11–19 years). When a patient had more than one measurement in any age group, the average was calculated for that range.

Descriptive statistics were calculated for each age group and presented as boxplots. Measurements across age groups were compared using one-way analysis of variance (ANOVA) with StataSE 14.0 software (StataCorp, College Station, USA). The results were considered significant when p<0.05. Additionally, smoothed curves for weight, height, and BMI by sex and age were generated using all available measurements. Generalized additive models for location, scale and shape (GAMLSS)^11^ were applied to model distributions beyond normality, using fractional polynomials of second or third order. Random effects were included to account for repeated measures from the same individual. The best models for each case were selected based on the lowest values for the generalized Akaike information criterion (GAIC). These calculations were performed using the *gamlss* library in R version 4.3.2 (the R Foundation).

## RESULTS

We identified 29 patients with DGS confirmed by genetic testing, including 2774 anthropometric measurements in total. After averaging repeated measurements within each age group, 961 measurements were included for analysis. Their demographic data are summarized in Table 1. Briefly, most of them were born with >3 kg, most had a suspected diagnosis before 1 year of age, had an associated congenital heart disease, were orally fed, and were followed up for a mean of 9 years.

**Table 1:** Demographic and clinical characteristics of patients with DiGeorge syndrome followed up at a tertiary-care referral hospital between 2009 and 2024 (n=29).

Characteristics	Results
Follow-up time (years)	9 [5.3]
Male gender	15 (51)
Birth weight	
<2 kg	1 (3)
2–2.5 kg	1 (3)
2.5–3 kg	4 (13)
>3 kg	17 (58)
Unknown	6 (20)
Age of suspected diagnosis	
<6 months	7 (24)
6 months–1 year	7 (24)
>1 year	7 (24)
Unknown	8 (27)
Congenital heart disease	
Ventricular septal defect	12 (41)
Tetralogy of Fallot	8 (27)
Multiple or complex heart disease	14 (48)
No heart disease	3 (10)
Thymus	
Absent	10 (34)
Present	7 (24)
Unknown	12 (41)
Hypocalcemia	
Absent	12 (41)
Present	12 (41)
Unknown	5 (17)
Lymphopenia (<2500)	
Absent	7 (37)
Present	12 (63)
Immunoglobulin replacement therapy	9 (31)
Nutrition route	
Oral	22 (78)
Enteral	6 (21)
Unknown	1 (3)
Chronic use of oral corticosteroids	3 (10)

Legend: Data are described as frequency (percentage) or mean [standard deviation].

**Figure 1:**
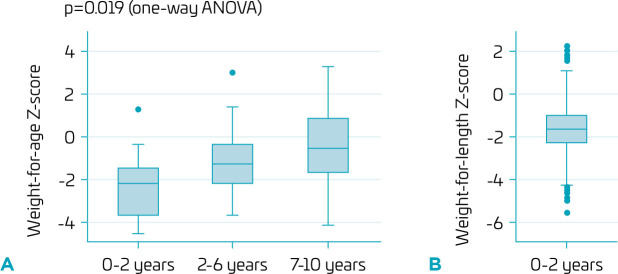
Weight-for-age (W/A, A) and weight-for-length (W/L, B) z-scores by age groups of patients with DiGeorge syndrome followed up in a tertiary-care referral hospital.

In the first 2 years of life, 50% of all W/A measurements were below a z-score of -2 (low W/A). After 2 years of age, the median W/A ranged between -2 and 0 (appropriate W/A), as shown in Figure 1A. There was a significant increase in W/A over time (p=0.019). W/L was below -2 (low W/L) in about 30% of patients (Figure 1B).

**Figure 2:**
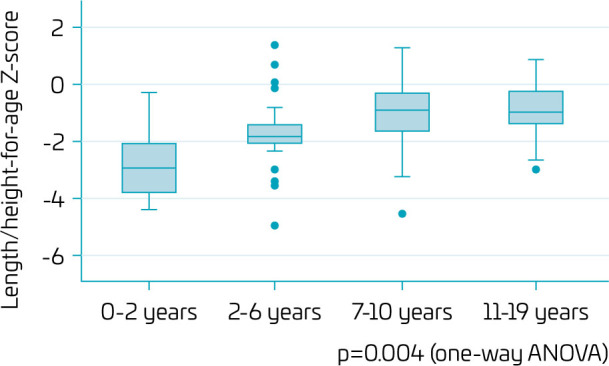
Length/height-for-age z-scores (LH/A) by age groups of patients with DiGeorge syndrome followed up in a tertiary-care referral hospital.

Regarding LH/A during the first 2 years of life, 75% were categorized as short for age (z-score below -2). From ages 2–19 years, median LH/A values were appropriate (z-score between -2 and 0), indicating catch-up, according to Figure 2. Over time, there was a significant increase in LH/A (p=0.004).

**Figure 3:**
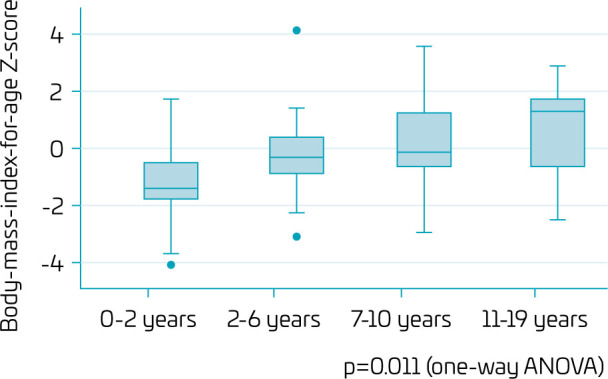
Body mass index (BMI)-for-age z-scores (BMI/A) by age groups of patients with DiGeorge syndrome followed up in a tertiary-care referral hospital.

Although almost 25% of the patients were wasted (BMI/A z-score below -2) before 2 years of age, there was a significant increase over time (p=0.011), and the median BMI/A was normal between 2 and 10 years of age. Afterward, the median BMI/A was above +1, indicating excess weight. Approximately 30% of the adolescents were overweight, and 25% were obese. Of note, between 11 and 19 years of age, BMI/A varied widely, possibly reflecting the DGS’s severity spectrum (Figure 3).

**Figure 4:**
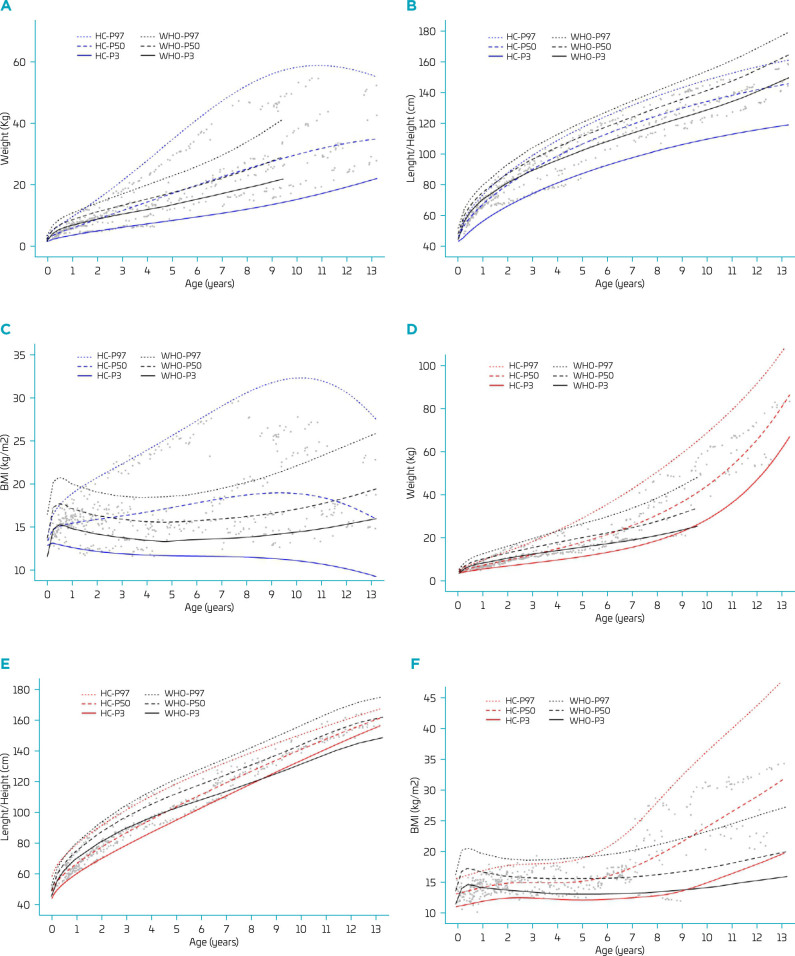
Smoothed curves of weight for age (W/A, A and D), length/height for age (LH/A, B and E), and body mass index-for-age (BMI/A, C and F) in males and females, respectively.

Legend: HC, this study; WHO, World Health Organization; P97, 97^th^ percentile; P50, 50^th^ percentile; P3, 3^rd^ percentile.

The estimated percentiles for weight, length/height, and BMI were also compared with the WHO curves, as indicated in Figure 4. Briefly, weight varied widely in boys and less in girls but was above the WHO standard/reference in the long term. Length/height was lower than the WHO reference/standard in boys, but not in girls. Finally, the BMI curves were grossly distorted by a wide variation in both boys and girls. Boys had values far above and below the normal limits, whereas girls mostly had values within the normal range or above the upper limit.

## DISCUSSION

This study provides a detailed analysis of growth trajectories in Brazilian children and adolescents with DGS, focusing on three distinct phases: impaired growth during early childhood, catch-up growth in preschool and school years, and a trend toward overweight or obesity in adolescence.^5,6^

One important observation is the fact that the proportion of children with a low W/L (thin, ~30%) and a low BMI/A (wasted, ~25%) before the age of 2 was lower than the proportion of those with low W/A (light, ~70%) and low LH/A (short, ~75%), suggesting that many children may have been born small for gestational age (SGA) but proportionate, having a low W/A but adequate W/L and BMI/A. The impaired growth observed in early childhood can be attributed to factors such as congenital heart disease, feeding difficulties, hypocalcemia, recurrent infections, and immune deficiencies commonly associated with DGS.^3^ Approximately 50% of our cohort exhibited severe growth restrictions (below the z-score -2) during the first 2 years of life. These results are consistent with findings from Tarquinio et al.^12^ who reported similar growth patterns in 25–50% of children with DGS. This emphasizes the importance of early interventions, such as nutritional support and management of comorbidities, to address growth deficits in this vulnerable population.

Catch-up growth observed after 2 years of age suggests the effectiveness of multidisciplinary care, including nutritional optimization and correction of congenital anomalies. Improvements in LH/A were noted, with most patients recovering to z-scores between -2 and 0 by 6 years of age. This pattern of growth recovery, reported in other studies, may be attributed to enhanced caloric intake and tailored medical management.^13^ However, it is worth noting that growth recovery may be influenced by genetic factors, as suggested by studies linking parental height to the growth trajectories of children with DGS.

Despite initial growth improvements, a significant proportion of patients developed overweight or obesity during adolescence. BMI/A transitioned from normal ranges (between -2 and 0) during childhood to above-average levels (z-scores between +1 and +2) in adolescence, with 30% classified as overweight and 25% as obese. These findings mirror those from Chilean and Italian cohorts, where obesity became prevalent during adolescence and adulthood.^6,7^ Brazil’s ongoing nutrition transition, characterized by increased availability of ultra-processed foods,^13^ may exacerbate this trend. Continuous monitoring and proactive interventions, such as dietary counseling and physical activity promotion, are essential to mitigate long-term metabolic risks.

The study also revealed sex-based differences in growth patterns. Boys exhibited greater variability in weight and height compared to WHO standards, while girls were more likely to exhibit normal or above-average BMI. These discrepancies suggest potential influences of hormonal, biological, or environmental factors specific to the Brazilian cohort, warranting further investigation.

Nutritional issues, including intestinal dysfunctions such as altered mucosal histology and microbiota composition, may also contribute to the observed growth challenges in DGS.^14^ These factors could hinder nutrient absorption and exacerbate weight variability, emphasizing the need for a holistic approach to patient management.

This study underscores the need for continuous and individualized care in patients with DGS:

a) addressing malnutrition in the first 2 years of life through optimized nutritional support and medical care;
b) implementing strategies to prevent excessive weight gain in adolescence, such as promoting healthy eating habits and physical activity; and
c) lifelong anthropometric monitoring to guide patient management and prevent complications associated with both undernutrition and obesity.

This study has several limitations. First, the small sample size from a single tertiary referral service and the retrospective design may limit the generalizability of findings and introduce potential biases. Second, anthropometric measurements were conducted by different professionals, though within a trained team in the same healthcare service. Third, patients with severe presentations who died at an early age were not included, potentially skewing the data toward less severe cases. Fourth, potential growth-determining factors were not examined in this study. Fifth, the stage of pubertal development was not analyzed, which may influence nutritional status. All patients with DGS are followed up in this service, regardless of having or not any type of immunodeficiency.

Future research can (a) explore the mechanisms underlying obesity in DGS, including genetic, hormonal, and environmental factors; (b) investigate the effectiveness of tailored interventions, such as nutritional programs and physical activity regimens, in preventing long-term complications; and (c) conduct multicenter studies with larger, diverse cohorts to enhance the understanding of growth patterns across different cultural and socioeconomic settings.

In conclusion, this study highlights the dynamic growth patterns in Brazilian children and adolescents with DGS, characterized by early growth impairments, subsequent catch-up growth, and a rising prevalence of overweight and obesity in adolescence. These findings emphasize the importance of early interventions, continuous monitoring, and comprehensive care to improve long-term health outcomes in this population.

## Data Availability

The database that originated the article is available with the corresponding author.
